# The SWIM study: Ethnic minority women's ideas and preferences for a tailored intervention to promote national cancer screening programmes—A qualitative interview study

**DOI:** 10.1111/hex.13309

**Published:** 2021-07-07

**Authors:** Camilla Rahr Tatari, Berit Andersen, Trine Brogaard, Sara Badre‐Esfahani, Negin Jaafar, Pia Kirkegaard

**Affiliations:** ^1^ Department of Public Health Programmes Randers Regional Hospital Randers Denmark; ^2^ University Research Clinic for Cancer Screening Department of Clinical Medicine Aarhus University Aarhus Denmark; ^3^ Laegerne i Gellerup Brabrand Denmark; ^4^ Department of Gynaecology and Obstetrics Aarhus University Hospital Aarhus Denmark

**Keywords:** decision making, Denmark, early detection of cancer, emigrants and immigrants, ethnic groups, health status disparities, mass screening, minority health, preventive health services, qualitative research

## Abstract

**Background:**

Ethnic minority women from non‐Western countries are less likely than the native women to participate in screening programmes for cervical cancer, breast cancer and colorectal cancer. This social inequality can result in loss of possibility for prevention, delayed diagnosis and treatment and, ultimately, lower chance of survival. Developing a tailored intervention might be the solution to reduce social inequalities in cancer screening, and a key feature in intervention research is to consult the target group.

**Objective:**

To explore ethnic minority women's own ideas and preferences for a cancer screening intervention and identify their attitudes to different strategies.

**Methods:**

An interview study with five focus group interviews, two group interviews with an interpreter and three individual interviews. Thirty‐seven women from 10 non‐Western countries contributed to the study. The interviews were audio‐recorded and transcribed verbatim followed by a thematic analysis.

**Results:**

According to the women, a tailored intervention should focus on knowledge in the form of face‐to‐face teaching. The women further suggested information material in their own language with a simple, positive and concrete communication strategy. They would like to be involved in an awareness strategy and share the knowledge with their network.

**Conclusion:**

Ethnic minority women were interested in a tailored intervention, and they were keen to contribute with ideas and preferences. The findings emphasized the potential of a tailored intervention with specific suggestions to the content when attempting to reduce inequality in cancer screening participation.

**Patient or Public Contribution:**

Minority women were involved in the interview study.

## 1 BACKGROUND

The purpose of offering organized cancer screening programmes for cervical cancer, breast cancer and colorectal cancer is to detect cancer or precancerous lesions before symptoms appear.^1^, ^2^, ^3^ Many Western countries including Denmark have implemented cancer screening programmes but in most cases ethnic minority women are less likely to participate in the screening programmes compared with the native women.^4^, ^5^, ^6^, ^7^, ^8^, ^9^, ^10^ Non‐participation can result in higher incidence rates, delayed diagnosis and treatment and, in worst case, inferior survival.^11^, ^12^, ^13^ Therefore, there is a need to explore whether a tailored intervention could be the right instrument for reducing social inequality in cancer screening for ethnic minority women.

Kreuter and Skinner offer a definition of a tailored intervention as ‘any combination of information or change strategies intended to reach one specific person based on characteristics that are unique to that person, related to the outcome of interest, and have derived from an individual assessment’.^14^ Our study adopts this definition except with a group perspective instead of an individual perspective. Some screening interventions for ethnic minority women (including strategies regarding reminders, accessibility and personal contact) have demonstrated improvements in knowledge, intentions to get screened and participation, but the evidence remains limited.^15^, ^16^, ^17^

Involvement of the target group may be the key to identifying strategies for increasing screening participation.^18^ Accordingly, we initiated the ‘Screening for Women with IMmigrant background’ (SWIM) study to interact with the target population and explore whether and how targeted interventions can secure informed decisions on screening participation among ethnic minority women. Recently, we found that barriers to screening among ethnic minority women included language difficulties, a general mistrust in the Danish health‐care system, unfamiliarity with disease prevention and early detection, and a belief that cancer screening is only relevant for people with symptoms and that cancer is incurable.^19^ This adds to literature which identifies common barriers such as low health literacy, stigma, fatalism, sexual morality, language barriers and lack of knowledge about cancer and screening offers.^20^, ^21^, ^22^, ^23^ These findings emphasized the potential for a tailored intervention for ethnic minority women as a way of reducing inequality in screening participation.

This study aimed to explore ethnic minority women's own ideas and preferences for a cancer screening intervention as well as their attitudes to different strategies attempted in similar settings.

## 2 METHODS

### 2.1 The Danish setting

All Danish citizens have free access to the three nationwide cancer screening programmes, follow‐up visits and treatment.^24^ All screening programmes have a reminder system and can be actively opted out. All communication and written material about the screening programmes are in Danish. Invitation letters contain a link to a web page with a brief description of cancer screening in English.

Women aged 23‐49 are invited to participate in cervical cancer screening every third year, and women aged 50‐64 are invited every fifth year. The invitation letters are sent by digital mail recommending the women to book an appointment at their general practitioner to have a cervical cytology specimen collected.^25^

The cumulative probability of participation in cervical cancer screening up to 2.9 years after receiving the invitation has been shown to be about 75% for Danish women and about 61% for non‐Western ethnic minority women.^7^ Women aged 50‐69 are offered biennial breast cancer screening. In the screening invitation, women are offered a pre‐booked mammography appointment at a screening hub.^26^ The participation rate is 80% for Danish women and 57% for non‐Western ethnic minority women in the Central Denmark Region.^8^ Finally, both men and women aged 50‐74 are offered biennial colorectal cancer screening using faecal immunochemical test self‐sample kits sent directly to their home. The men and women are invited to collect a faecal sample and mail it directly to a laboratory.^27^ The participation rate in this programme is 72% for Danish women and 56% for non‐Western ethnic minority women.^9^ By 1 January 2019, 14% of all immigrants and descendants composed the total Danish population, and non‐Western immigrants and descendants composed 8.8%. Most non‐Western immigrants and descendants are from Turkey, Syria and Iraq, followed by Lebanon, Pakistan, Bosnia‐Hercegovina, Iran, Somalia, Afghanistan and Vietnam. Immigrants from Turkey and Pakistan came in the 1960s for work while immigrants from the other countries mainly came as refugees or as part of family reunification programmes.^28^

### 2.2 Design, participants and recruitment

The study was designed as a qualitative study with non‐Western ethnic minority women recruited to the study through snowball sampling.^29^ Recruitment was done in different social societies in the socially deprived suburban area Gellerup in Denmark's second largest city, Aarhus. Gellerup has about 6000 citizens out of which 54% are unemployed and 81.3% are from non‐Western countries, the majority being immigrants from Somalia and the Middle East.^30^, ^31^

The participants were recruited according to a maximum variation sampling strategy^32^ including both first‐ and second‐generation ethnic minority women from non‐Western countries between 23 and 74 years old. A general practitioner in the suburban area (co‐author TBR) and the local society ‘the Neighbourhood Mothers’^33^ identified eligible participants. All participants were given verbal and written information about the study in either Danish or their native language and were asked to fill out a consent form in either Danish or their native language before contributing to the study's data collection. After each interview, the participants were offered a gift voucher worth 25 euros as an appreciation of their participation. A continuous assessment of information power during the collection period determined the number of participants.^34^

In total, 37 women from 10 non‐Western countries participated. Twenty of the participants had previously participated in minimum one of the cancer screening programmes at least once. The age of the participants ranged from 27 to 59 years old with a median of 39 years. Only two participants were more than 50 years old and currently eligible for screening for breast cancer and colorectal cancer. Thirty‐one of the participants had lived in Denmark for more than 10 years, 18 were married, ten were either unmarried/single or divorced, and nine did not state their marital status. Thirteen were employed or self‐employed, five were enrolled in education, 13 were unemployed, and six did not state their employment status. Sixteen had up to three children, while 13 had more than three children. For further details about the design, participants and recruitment strategy, please see Tatari et al 2020.^19^

### 2.3 Data collection

The data collection was based on semi‐structured focus group interviews, more structured group interviews with an interpreter and semi‐structured individual interviews with local key persons with a comprehensive knowledge about different cultures in the area. The authors designed an interview guide to assess specific themes while allowing the participants to communicate freely.^35^ All questions were checked for literacy level and potential ambiguity by women from the Neighbourhood Mothers and adjusted accordingly. The focus groups and individual interviews were undertaken in Danish with women who understood and spoke Danish. Women who did not speak Danish were interviewed with a tele‐interpreter. The phrasing of the questions was slightly modified according to the interview type and the participants' language skills. After conducting each interview, field notes and immediate impressions were compared and discussed among the moderators. The preliminary results were discussed among all authors to adjust the interview guide for the following interview(s). The data collection is described in detail elsewhere, including the interview guide.^19^

All interviews began with a short introduction to the aim of the study and ended with a debriefing during which the participants were given an extra opportunity to speak freely. The first part of the interviews focused on the participants' perceptions and knowledge about cancer and screening. The results of this part of the interviews were reported in a previous publication.^19^ The second part of the interviews was initiated with the open question ‘what is screening?’ in order to get the participants involved from the beginning and to encourage a dialogue open for questions. After the open question, one of the moderators gave a short presentation (~15 minutes) about cancer screening and its purpose using hand‐drawn graphics. As an example of the natural history of cancer, cervical cancer screening was presented mere thoroughly. After the presentation, the women were asked about their thoughts on the subject and ideas for content in a tailored intervention aiming to create awareness and make ethnic minority women take an active decision to participate or not. They were then asked for their views on two different types of HPV self‐sampling kits, which are not yet implemented in the Danish cervical cancer screening programme (Evalyn brush^36^ and Qvintip^37^ ) and a self‐sampling kit used in the Danish colorectal cancer screening programme (a faecal immunochemical test^38^). Finally, there was a discussion of different reminder strategies (general practitioner, SMS, digital mail and a letter) and drop‐in screening (mobile breast cancer screening unit and a monthly day at the health centre to be screened for cervical cancer without an appointment). This was followed by the showing of a short video available in different languages from the campaign ‘Say yes to screening for cervical cancer’ developed by the Region of Southern Denmark.^39^ This publication is based on the content of the second part of the conducted interviews.

Five focus group interviews were conducted, each with five to six participants. The mean interview length was 104 minutes (ranging from 81 to 130 minutes). Some focus groups had mixed participant origin and some had homogeneous origin. Two group interviews with an interpreter were conducted, one with two Middle Eastern participants (54 minutes) and one with four Somali participants (83 minutes). Three individual interviews with local key persons in the area and culture (Somali, Arab and South‐East Asian) were conducted with a mean interview length of 65 minutes (ranging from 55 to 77 minutes). The interviews were conducted in different venues within the local area according to the participants' wishes: a closed beauty salon, a meeting room in a community centre, own home and a meeting room at the local mall. All the interviews were audio‐recorded and transcribed verbatim by the first author, CRT. Data were collected from April 2019 to June 2019.

### 2.4 Data analysis

A thematic analysis was conducted, inspired by a phenomenological approach where the women's own ideas and preferences related to the phenomenon 'cancer screening' were explored.^40^, ^41^ The analyses were managed and documented using a text editor with the ability to track changes and add comments. In the first step, the audio recordings were played to obtain an overall impression of the material and the transcripts of the interviews were meticulously read. In step two, the analysis began with an open coding of the interviews to describe the content. This was followed by a search for patterns or themes across the data in step three. In step four, the themes were reviewed; and in step five, they were labelled. The preliminary analyses and suggestions for themes were discussed in between these steps with members of the Neighbourhood Mothers to validate the interpretations conducted by the authors. The analysis was a dialectical interaction between the steps mentioned before the results were final.

## 3 RESULTS

The following three main themes emerged from the data analyses: information and dialogue about cancer screening, tailored communication and accessibility.

### 3.1 Information and dialogue about cancer screening

Many of the participants expressed that they had an eye‐opening experience during the presentation of the cancer screening programmes, especially the part where the steps in cancer's natural history were explained and sketched. Several women stated that they knew nothing beforehand and now they understood why cancer screening was relevant: ‘We would like to know WHY it [cancer] appears and the reason for it [the cause of cancer]. Knowledge, it all comes down to knowledge… Knowledge is everything!’ (Focus group 4). They said that it worked well when the simple drawings were created right in front of them and they referred to the drawings when they had a question.

The women were passionately advocating for a group‐based intervention in the community with the opportunity for questions and face‐to‐face communication: ‘I'd rather come to it [screening] after something like this [short presentation]. Because I knew that cancer existed but this [points at the drawing of the natural history of cancer] I didn't know. It's something else when it's face to face, you have a better sense of what it's about and you can ask your questions. You can't do that to a letter or a video’ (Focus group 2). There was a consensus that the teacher should have a health professional background and be able to answer their questions during the cancer screening presentation.

The majority thought that cancer screening was a sensitive and private subject and presentations about cancer screening should be held for smaller gatherings or for participants who knew and trusted each other: ‘It can be embarrassing for some people. I mean, it's a private subject, so I'm thinking smaller gatherings. Most I know would prefer that… And maybe people from the same social network, because it's easier to talk to people you know’ (Focus group 4). The cancer screening presentation could be performed in local cultural societies as an extra item on their meeting agenda: ‘It [the screening presentation] must be in some kind of a forum, which is already set up, and then you invite yourself, so to speak, to give your presentation’ (Focus group 3). Others mentioned that it should be possible to book a teacher for home gatherings for smaller communities for women.

### 3.2 Tailored communication

The participants found the current written Danish information in the invitation letters to be overwhelming and hard to navigate. Instead, they wished for more clear and simple information preferably supported by oral presentations (as mentioned above) and visual communication: ‘That letter doesn't work for me – I'm missing something else like this [the short presentation]’ (Focus group 1).

Visual communication was preferred by nearly all the women, but at the same time, there was a need for balance. When communicating visually about cancer screening, it should be done neither patronizing nor offensive: ‘It should be drawings, pictures or an animated film. But it should not be offensive’ (Focus group 1). When presented with anatomically correct drawings of the abdomen as an example of visual communication, many of them reacted with disgust: ‘Oh, it's too much! It won't work; we don't want to see that’ (Focus group 1). The women suggested that the invitation should have more illustrations and less text to make it more comprehensible. Many suggested that the official cancer screening information was available in both Danish and in their native language in the invitation they received by digital mail. Some objected to this idea because they would feel offended, while others would think that it was a nice gesture for those who needed it. However, they could all agree on: ‘The biggest challenge here in the area is the language’ (Focus group 2).

The participants mentioned that for many people cancer was strongly associated with death. Specifically, it was mentioned that the word ‘cancer’ should be avoided or used as little as possible in a communication strategy. It was suggested that the communication should have a more hopeful and positive tone: ‘Get screened to avoid having your uterus removed… Like that, so there is a little hope in it. Screening is hope’. (Focus group 1). The women argued that it should be communicated clearly that cancer may be prevented or cured if it is detected early: ‘It's better to know it sooner so you can do something about it than knowing it too late’ (Focus group 5).

The women had many ideas regarding communication channels for a tailored intervention. This included local societies such as The Neighbourhood Mothers: ‘The Neighbourhood Mothers is a good place and there are many different people there; also some who just immigrated and don't know anything about this offer’ (Focus group 5). Also, religious societies were mentioned including the wife of the local imam. The women argued that different communication channels should be applied for young and old groups. The older generation used Facebook, while the younger generation was more active on Instagram, WhatsApp and YouTube.

Several mentioned that they had received leaflets about many different subjects like recycling or different health issues over the years, and they found it rewarding to keep themselves updated from a leaflet with illustrations and limited text. They suggested that after the screening presentation, the audience should receive a leaflet with the key messages and illustrations: ‘First you should tell about screening, what it is and why it's relevant, and then you should hand out a leaflet in their native language… and maybe some extras we can pass on’ (Focus group 3). It should be visible and available at the general practitioner's, the library or the local bazaar. It was mentioned many times that a campaign with leaflets and posters with both pictures and illustrations in the local area might be the best ways to create awareness about screening. The videos received mixed responses. During the playing of the videos on an iPad, many of the participants paid divided attention to their mobile phones instead of focusing on the video content. Overall, the women agreed that a video like that could be suitable to create awareness on social media but should not stand alone.

The women mentioned the pass‐it‐on strategy like a ripple effect: ‘If you make such a group as we sit here today and give them a basic course in cancer screening; then you have a group of people who will go out and pass on the important messages. That's what it's all about’ (Focus group 3). Some participants told about a bigger group of women in the area who do not get out much due to social control, health issues or old age. These women still got visits from their peers. The Neighbourhood Mothers was mentioned as an excellent way to start the ripple effect: ‘You should cooperate with the Neighbourhood Mothers; they have groups all over the country and they can also reach the isolated women. If you get the Neighbourhood Mothers on board, well, they talk to everyone’ (Focus group 3). Overall, the women were really supportive of a screening offer and wanted to get involved and contribute to the best of their ability and draw on their network.

### 3.3 Accessibility

All but two of the participants were too young to be invited to breast cancer screening and colorectal cancer screening at the time of the interview but they were keen to discuss their attitude to these screening programmes and whether they would participate when they reached the screening age. They were positive about the faecal immunochemical test self‐sample kits for colorectal cancer screening and thought it would be quick and easy to participate. It was mentioned by many participants that the older generation might not be as positive about it as they were: ‘Sending their faeces may be transgressive for the older generation. They can't understand why researchers want to mess with it [everyone laughs]’ (Focus group 1). Some were willing to pass on the message about screening to their parents.

In the breast cancer screening programme, the women did not see transportation to the screening clinic as a barrier. They did not get the idea of a mobile screening bus unit and they were not interested in arranged transportation options or other versions of drop‐in screening. The two HPV self‐sampling kits received mixed reactions. A few were excited about the kits and argued that a self‐sampling kit was a relevant opportunity for vulnerable women because the gynaecological examination would make more sense to them if they had a positive test result: ‘With that one, I think you've caught the right audience – 100%. There are many people who have been waiting for that one’ (Focus group 3). Most preferred to go to their general practitioner because they questioned their own ability to use the self‐sampling kit. Others were very positive about the self‐sampling kits until they heard that a positive test result would require a gynaecological examination anyway. With this information in mind, they said they would rather go to their general practitioner to begin with.

## 4 DISCUSSION

Overall, it was crucial to the women that a tailored intervention focused on information about why cancer screening is beneficial and important. The women were particularly excited about the possibility for interaction with a health‐care professional in the form of face‐to‐face teaching in smaller groups with an opportunity to ask questions. Furthermore, the women suggested screening information in their own language, simple and concrete with pictures and illustrations and with a positive tone including the message that cancer can be cured and screening is for asymptomatic people. In addition, the women argued that women who had received the presentation about why screening is important would share the knowledge about the screening offers with their network. A leaflet would then work as a reminder and make it easy to pass on the message about why screening is important to the more isolated and vulnerable women.

### 4.1 Strengths and limitations

Focus groups were the main data source in the study. The participants confirmed or challenged each other's points of view on a tailored intervention which allowed us to gain a complex and varied picture of the ethnic minority women's ideas, needs and preferences. In addition to the focus groups, we also collected data from individual interviews and interviews with an interpreter to collect and verify as many relevant perspectives as possible. However, the fact that interview guides were not translated and administered by a same language researcher in the interviews with women who did not understand Danish could be considered a limitation of the study.

We consider it a strength of the study that we recruited participants from 10 different countries. Twelve out of 37 participants had a Somali background which at first may seem like an over‐representation. However, in Gellerup the inhabitants are predominantly from the Middle East and Somalia and the study population reflected the area. Overall, the participants comprised a highly diverse group with respect to ethnicity, years of stay in Denmark, employment status, marital status and number of children. Another strength was the participants' opportunity to choose the location for the interview themselves. This was decided to ensure a setting where the participants should feel at home in a relaxed atmosphere.

A limitation of the study was that only a few of the participants were more than 50 years old due to the fact that elderly women with ethnic minority background were hard to recruit. A study about Somali immigrant women in the United States showed that younger Somali women (aged 20‐35) accepted screening and preventive care whereas Somali women above 36 years of age were more likely to state that they did not see health care as preventive and only wanted to go to see their doctor when they felt sick, because cancer was not preventable but God's will.^42^ The women in our study argued that the younger generation should encourage screening uptake and help the older generation who had problems navigating the health‐care system in the host country. This indicates that the younger generation may play a significant role in supporting the older generation and are willing to do so.

Most of the participants were recruited knowing they would receive a gift voucher worth 25 euro. It could be argued that this could lead to a selection bias as women with a low income would be more inclined to participate than women with a high income. However, we did not observe this bias in the sample and some participants even declined the gift voucher saying they enjoyed learning about an important topic and should not be compensated for their time. When we suggested they could give the voucher to someone they loved or cared about, some of the reluctant participants accepted the voucher. Thus, a gift voucher was not the primary incentive to contribute to the study.

There are often more similarities than differences between ethnic groups of immigrant women regarding participation in screening and they experience considerable stress associated with their minority status which in itself hinders participation.^43^, ^44^, ^45^ However, the findings in our study may mostly reflect the general view of ethnic minority women predominantly from the Middle East and Somalia in deprived areas in a health‐care system which is publicly funded and in other ways similar to Denmark in terms of number of immigrants, social inequalities, etc

### 4.2 Discussion of findings

A systematic review from 2010 published guidelines for adapting health‐promoting interventions for ethnic minority communities.^45^ A key principle was to ‘use community resources to increase intervention accessibility’. In line with this principle, the women in our study pointed out many different networks and key persons in the area as well as social media as effective ways to communicate the message. A Swedish study showed that women from, for example, the Middle East and Somalia wanted communication by local actors (eg doulas) who shared their cultural background/minority status.^46^ Some women in our study expressed this desire too, but most were more concerned about the professional integrity of the teacher than the ethnic background, despite the fact that many of them expressed mistrust in Danish medical professionals. It was important to them that the teacher had been vouched for by local actors in the community. The Swedish study showed that information about screening should be provided to women below the screening age, to prepare them when their screening invitation would arrive a few years later. Similarly, the women in our study argued that teaching about screening for breast cancer and colorectal cancer would prepare them for the invitations to come. They were keen to disseminate their newly acquired knowledge and communicate to elderly women in the screening age (mothers, aunts, friends of the family) what screening is about. They acknowledged the role of each individual woman in conveying the message about screening to women who did not or could not attend the teaching sessions, for instance elderly women or vulnerable women.

An integrative review about facilitators for screening among Somali immigrant women showed the strength of collaboration between community partners in engaging the target group in screening.^47^ It suggested that community members should be trained as health outreach workers to create an atmosphere of trust, and initiatives should involve training of community health workers when planning and executing education programmes. This supports the findings in our study. Two studies from Norway showed that community‐based education presented in native language was important but they emphasized that communication should be orally presented.^20^, ^48^ The women in our study also suggested material (leaflets and posters) written in native language but emphasized that it was only a supplement to oral presentations.

A review from 2017 showed that interventions which were found to successfully improve participation in screening included pre‐screening reminders, endorsement from the general practitioner and more personalized reminders for non‐participants.^15^ The women in our study did not suggest any of these strategies. They made it clear to us that an intervention should focus on knowledge and awareness through education and communication strategies explaining why screening is important and how screening creates hope for early and better treatment. A possible explanation of the discrepancy between the review and the findings in our study might be that the review focused on underserved populations in general while this study focused on ethnic minority women only.

In our study, the HPV self‐sampling kits received mixed reactions; some preferred screening by the general practitioner while others had been waiting for the opportunity to conduct self‐sampling. A Danish trial conducted among non‐participants showed a positive effect on participation when non‐Western immigrant women were offered the opportunity of self‐sampling.^49^ However, when non‐Western immigrants had the choice between self‐sampling and general practitioner‐based screening, a small majority chose the latter over self‐sampling (17.7% vs. 14.9%). This finding was similar to the findings in our study and several focus group studies.^50^, ^51^, ^52^, ^53^ Consistent with these studies, our study found mistrust in the ability to conduct proper self‐sampling to be a barrier. Furthermore, mistrust in the Danish health‐care system has shown to be a barrier for participation in the national screening programmes.^19^ When one does not trust the health‐care system, reminders might not be enough. In this situation, increasing knowledge, involvement and awareness may be a better strategy to enable ethnic minority women to make an informed decision on whether to participate in cancer screening.

## 5 CONCLUSION AND IMPLICATIONS

This study indicates that a tailored intervention offers potential for increasing participation in cancer screening programmes for ethnic minority women. The ethnic minority women in the study were interested in a tailored intervention and had strong views on what it should contain: an educational strategy with group teaching sessions held by a health‐care professional, a communication strategy with information in their native language, a positive tone, a clear message, illustrations, a strategy to increase awareness about the screening offer and a strategy to involve the women in actively spreading the word about the screening offer in their network.

The findings indicate the potential and importance of a tailored intervention with specific suggestions to the content in the attempt to reduce social inequality in screening participation.

## CONFLICT OF INTEREST

The authors have no conflict of interest to declare.

## AUTHORS' CONTRIBUTIONS

PK and BA conceived the original idea and designed the details of the study together with CRT. CRT recruited the participants in collaboration with TBR and the Neighbourhood Mothers. CRT, PK and SBE conducted the data collection. The initial coding was conducted by CRT and PK. All authors were involved in the final analyses and discussions. CRT made the final write‐up of the manuscript. All authors read and approved the final manuscript.

## ETHICAL APPROVAL

According to EU's General Data Protection Regulation (article 30), the project was listed at the record of processing activities for research projects in Central Denmark Region (journal no. 1‐16‐02‐368‐19). The study followed the principles from the Statements on Ethics of the American Anthropological Association.^54^ In accordance with Danish legislation (ie the Act on Research Ethics Review of Health), further ethical approval was not required.^55^ Both written and verbal consent was obtained from all study participants.

## CONSENT FOR PUBLICATION

All participants gave consent for publication.

## Data Availability

The datasets used and/or analysed during the current study are available from the corresponding author upon reasonable request.
